# Cesarean Section in the Delivery Room: An Exploration of the Viewpoint of Midwives, Anaesthesiologists, and Obstetricians

**DOI:** 10.1155/2018/1017572

**Published:** 2018-09-27

**Authors:** Jansegers Jolien, Jacquemyn Yves

**Affiliations:** Antwerp University Hospital (UZA) and Antwerp University (UA) – ASTARC, Belgium

## Abstract

**Aim:**

To explore the attitude and vision of midwives, anaesthesiologists, and obstetricians concerning a dedicated operating room for cesarean sections within the delivery ward versus cesarean sections within the general operating room.

**Method:**

A descriptive qualitative study using a constructive paradigm. Face-to-face semistructured interviews were performed in 3 different hospitals, one without operating theatre within the delivery ward, one with a recently built cesarean section room within the delivery ward, and one with a long time tradition of cesarean section in the delivery room. Interviews have been analysed thematically.

**Results:**

Three themes have been identified: organization, role of the midwife, and safety. Although identical protocols for the degree of emergency of a cesarean section are used, infrastructure and daily practice differ between hospitals. Logistic support, medical and midwife staffing, and hospital infrastructure are systematically mentioned as needing improvement. Realizing cesarean section within the delivery ward was considered as an improvement for the patient's experience. Midwives need a clear and new job description and delineation and mention a lack of formal education to assist surgical procedures. To increase patient safety continuous education and communication are considered necessary.

**Conclusion:**

A detailed job description and education of all those involved in cesarean section at the delivery ward are necessary to improve patient safety. Patient experience is improved, but our knowledge on this is hampered by lack of studies.

## 1. Introduction

Cesarean section rate is rising all over the world [1]. The process of becoming parents is a major life event of multidimensional, complex, and unique character, influenced by the surroundings where it takes place and a continuous search to improve this experience is necessary [2]. A reorganization of traditional obstetrics in a multidisciplinary context including a change of location can be part of this amelioration.

An essential part of improving the experience of cesarean section consists in avoiding separation of mother and child including skin to skin contact immediately after birth in the operating theatre [3, 4]. Frederick et al. concluded that women highly appreciated not being separated from the child during and after cesarean section [5].

A dedicated operating room for cesarean section within the delivery ward can help implement continuous skin to skin contact and improve patient experience; such a specified cesarean section room is not connected to the main operating block [6]. A specialized cesarean section operating room not connected to the central surgical unit is not widely available. While constructing a new perinatal unit we performed a small survey in 2015; only 3 of 88 (3.4%) delivery units in Flanders (Belgium) had this available; the Belgian Association of Regional Association also was not able to provide a general advice on this as during development of their guideline major discussions arose, resulting in a very general guideline stating “the location for an elective or urgent cesarean section is variable form one hospital to the other” [7]. Furthermore until 2018 midwives did not receive systematic training in assisting anesthesiologists or gynaecologists during cesarean section in our country; since 2018 by law this is made part of the formation to become a midwife. This situation has prompted us to further explore the actual views of anesthesiologists and midwives on cesarean section in the delivery room.

The place where delivery takes place influences not only the way the mother-to-be lives through this major life event, but also the attitude of midwives and thus should be taken into consideration when trying to improve the care given to parturient women [8]. On the other side for an anesthesiologist leaving the trusted environment of the operating theatre can be a threatening experience or can provoke uncertainty.

The aim of the current research is to analyse the views of midwives, obstetricians, and anaesthesiologists on performing cesarean sections outside the main operating block and in the delivery ward.

## 2. Methods

### 2.1. Design

We performed descriptive qualitative research using a constructive paradigm. The paradigm states that any person has his/her own views and interpretation of reality; no interpretation is considered superior to another. Face-to-face semistructured interviews were performed and recorded. The study population consisted of midwives, gynaecologists, and obstetricians from three hospitals in the region of Antwerp, Belgium. All 3 hospitals are comparable as to the number of deliveries, between 1500 and 2000 per year. The hospitals are different for the infrastructure related to a cesarean section room in the delivery ward. The first hospital (Sint Augustinus Hospital) has a long-standing tradition of cesarean sections in the delivery room; the second hospital (Antwerp University Hospital (UZA)) had started performing cesarean sections in a new operating theatre in the delivery suit 6 months before our study; the third hospital (Middelheimziekenhuis) has no operating theatre in the delivery room; all cesarean sections are performed in the main operating theatre. In every hospital at least one midwife, one anaesthesiologist, and one gynaecologist have been interviewed. Every interview followed a previously determined scenario including 3 topics: cesarean section, location, and working experience. Respondents in each hospital have been selected by sending an email to all midwives, obstetricians, and anaesthesiologists working at the obstetric department. Data collection was started in December 2016 and ended in April 2017. The scenario for the semistructured interviews included after presentation of the interviewer (JJ) a short explanation that the study aimed at describing the viewpoint of professionals regarding cesarean section within the delivery ward. First basic demographic questions were asked (which hospital do you work in, which department, function, and years of experience within the job). Then the following were asked: what the local standard protocol for cesarean sections was and what the participant's personal experience with this was. In case an operating theatre was present in the delivery ward we questioned when it was used (always, mostly, only in emergencies, or any other description), and whether only sections were done or also other procedures. Next questions were on personal work experience considering advantages and disadvantages of this location, personal experience in cooperating with other disciplines, any other remark or recommendation one would like to make, and how the respondent thinks pregnant women and their family think about the location.

### 2.2. Analysis

All interviews were recorded electronically and coded. Analysis was based on a thematic approach. The interviews were transcribed and code book was developed. To avoid bias the first interview was coded not only by the researcher but also by an independent other; the code book was developed in consensus and is given in Table 1.

Ethical committees of each hospital have reviewed and approved this research. Written informed consent was obtained from each respondent before the interview.

## 3. Results

Qualitative thematic analysis resulted in three main themes (see Table 1): organization, role of the midwife, and safety.

Four anaesthesiologists, 3 obstetricians, and 3 midwives have been interviewed. Demographic data are listed in Table 2. Analysis revealed three major themes: organization, role and function of the midwife, and safety.

In both hospitals that perform cesarean sections in the delivery suit organization and location were similar. The dedicated cesarean section room is next to the delivery room and connected to a neonatal reanimation room. After cesarean section the patient is observed for one hour in a room with maternal monitoring. Cesarean sections are coded according to emergency degree by colours: red, orange, yellow, and green from highly urgent to elective. The system with colours is highly appreciated as it improves communication. Quote: “we have introduced this colour system as an urgent cesarean section was quite differently experienced by obstetricians versus anaesthesiologists; code red means immediately, that is clear for everyone, no delay” (an anesthesiologist). In the hospital that does not perform cesarean sections in the delivery ward, this was a specific choice because the anaesthesiologists considered it an unsafe practice.

In every interview it was clear that medical and midwife staffing was crucial and difficult because it was not reported by the hospital. Quote: “first it was decided that a cesarean section room was going to be built in the new delivery ward, but then hospital management did not allow for extra midwives or anaesthesiologists” (a midwife). All respondents mentioned that extra staffing is necessary. Dedicated anaesthesiologists for the delivery ward are considered necessary especially in case of an emergency. Quote: “it should be possible to have an anaesthesiologist available all day and an extra midwife for planned an unplanned cesarean sections, but in our context this is financially impossible” (anaesthesiologist). The location of the obstetric department and the distance to the general operating block are considered crucial factors for patient safety. Quote: “in my opinion a cesarean section room can be organised autonomically but it should be localised next to the central operating block” (anaesthesiologist). On the other hand, others considered having an operating theatre within the maternity unit safer when the distance with a central operating block is long “I think in all hospitals with the distance between the maternity and operating block is more than 100 m, there should be an operating theatre within the maternity” (anaesthesiologist).

All respondents consider a dedicated cesarean section room within the delivery ward as a positive experience for patient as mother and child are not separated and can stay together all the time; also the recovery period in which mother and child are often not together can be deleted; this improves skin-to-skin contact, first breastfeeding, and mother-child bounding. Quote: “all patients are happy, especially that they do not have to go to the recovery room and they are together with the baby continuously now, also the partner can stay with the baby and a woman all the time, patient is really appreciate that we perform cesarean sections here in our department” (a midwife). In the hospitals where it is not possible to perform a cesarean section in the delivery ward alternative ways to improve the experience are looked for; in the recovery room, an isolated space where the mother can stay with her baby was realized but financial limitations made it impossible for a midwife to stay with the mother.

It is clear that midwives do not feel prepared from the formation to assist in operating theatre cesarean sections; in one of the hospitals where cesarean sections were only recently performed at the maternity unit, operating nurses were still assisting and this was considered as a source of frustration; obstetricians and anaesthesiologists know exactly what to do in the operating theatre; for the midwives this was a new experience and their exact job and responsibility was not clearly defined. Tension arises when an operating nurse enters the cesarean section room. Quote: “midwives are not nurses and have no experience with working in operating room, they have not received any formation for that and during interventions really feel the difference between an operating nurse and a midwife” (gynaecologist); “we need training more than we have received until now; if anything goes wrong I would not know what to do; I do not feel certainly enough to stand alone as a midwife in the operating together” (midwife). Midwives feel that it is difficult on one hand to be a technical person and assisting the anaesthesiologists or the obstetrician and on the other hand to be present for the parents and taking care of the baby. Training and communication are mentioned as highly important especially in emergency situations.

Logistics are also considered of high importance; after each cesarean section all the materials should be checked and frequently discussions rise regarding who has to perform this; anaesthesiologists that are not used to working on the obstetric department mentioned that they feel unsafe in a room where they do not feel at home as in their usual operating theatre.

## 4. Discussion

From these interviews it becomes clear that introducing cesarean sections into the delivery ward makes it necessary that midwives receive extra training and formation to assist surgery as in Belgium, and most countries, a midwife is not a nurse. In general in the formation of midwives the physiology of labor and delivery is considered crucial; it can be questioned whether in a changing world it would not be better to train midwives systematically for assisting cesarean sections. The competencies that midwives should achieve are changing [9]. Actually the changing practice creates an enlarging gap between theory at school and practice in the delivery room [10].

Anaesthesiologists are much more critical when it comes to patient safety; midwives and obstetricians have more interest in the positive experience of the patient. This demonstrates the technical medical view of anaesthesiologists versus the more physiologic and normal medical view of midwives and obstetricians. To realize safety teamwork is of the utmost importance, especially in obstetric care [11]. This can be optimalized by team training and education especially simulation [12].

Literature on dedicated cesarean section room within a delivery unit is scarce. Graham et al. describe the experience in an academic hospital where patients planned for elective cesarean experienced waiting times and difficulty in transport to the operating theatre [2]. To improve the experience patients were prepared for surgery in the same room they would stay in after surgery; family could stay with them and they no longer stayed in a recovery room avoiding separation of mother and child. This changing policy was generally highly appreciated by the patients

Kasagi et al. describe the start of an operating theatre in a perinatal unit, away from the central operating block, a situation comparable to the hospital as we describe in this paper [6]. Before starting surgery in this new unit formation was given to the medical staff and the midwives by an experienced anaesthesiologist and operating nurse; in this hospital logistics and extra medical and midwife staff were provided before implementing the change. The interval between the decision to perform a cesarean section and birth of the baby is diminished. Kasagi et al. conclude that the lack of staff makes this dedicated cesarean section room not available for 24 hours. Patients find it less stressful.

In our descriptive study the same limitations are mentioned: lack of staff and lack of training; also the same advantages are mentioned, especially the positive experience by the patient.

Our study is limited by its quantitative and descriptive setup and by the small number of interviews. We do not know whether the opinions of anaesthesiologists, obstetricians, and midwives in our sample can be generalised but they do seem to be in the same line as what is published. We did not interview patients or family.

Discussions on the safety of the concept of an isolated cesarean section room seem to be frequent between anaesthesiologists and obstetricians; it is remarkable that almost no studies are available [13–15].

When introducing a dedicated operating room for cesarean sections within the delivery ward we recommend that this process be developed together with anesthesiologists and midwives from the very start. As concerns the midwives an intense coaching by trained operating nurses who progressively leave responsibility to the midwives is advised. A rotating period in which midwives do perform basic practice in the general operating room, in case this was lacking during basic training, is considered worthwhile. A dedicated team of obstetrics anesthesiologists would most probably be the ideal situation, but this can be difficult to realize in most centres. Anyhow involving anesthesiologists in the organization of the delivery ward operating theatre so they feel at ease with the location and material is necessary. Regular phantom training with the multidisciplinary team can also increase the ease of working.

## 5. Conclusion

Advantages for the patient and family are often mentioned as arguments in favour of cesarean section within the delivery department; safety issues are unclear. Transposing surgery from the operating theatre to the delivery ward necessitates a new competency profile of the midwife. More systematic research on this subject is necessary to guide us further in the organization of obstetrics in hospitals.

## Figures and Tables

**Table 1:** Codetree.

Descriptive codes	Professional experience
	Use of labor ward operating theatre
	Role of the midwife
	Interprofessional cooperation
	Advantages of labor ward operating theatre
	Disadvantages of labor ward operating theatre
	Recommendation for use of labor ward operating theatre
	Other remarks on labor ward operating theatre
	Location of the general operating theatre
	Quality and safety

Interpretative codes	Role of the midwife
	Role of the anesthesiologists
	Interprofessional cooperation
	Use of labor ward operating theatre
	Advantages and disadvantages of labor ward operating theatre
	Other remarks on labor ward operating theatre
	Patient and work safety

Subthemes	Use of labor ward operating theatre
	Advantages and disadvantages of labor ward operating theatre
	Remarks and recommendations on the use of this space
	Role of the midwife
	Interprofessional cooperation
	Working safely

Themes	Role of the midwife
	Organization
	Safety

**Table 2:** Demographic characteristics of respondents.

**Function**	**Sex**	**Years of professional experience**
Midwife	Female	7

Midwife	Female	27

Midwife	Female	25

Obstetrician	Female	23

Obstetrician	Male	20

Obstetrician	Female	21

Anesthesiologist	Female	27

Anesthesiologist	Male	3

Anesthesiologist	Female	10

Anesthesiologist	Male	35
